# Books and Magazines

**Published:** 1907-10

**Authors:** 


### Books and Magazines.

A TEXT-BOOK ON URIC ACID AND ITS CONGENERS, with Special Reference to Its Physical and Chemical Properties, Its Metabolism and Accumulation in the Organism, Together with the Disease Processes Arising Therefrom and Their Etiological Therapy. For Medical Students and Practitioners. By George Abner Gilbert, M. D. First edition, 1907: Danbury Medical Printing Co., Danbury, Connecticut, U. S. A.

This is a cloth bound book, fully indexed, and contains about 300 pages. It retails for $3. The author has for many years made a special study of the uric acid subject, and has embodied in this work the results of his investigations. Nowhere in the English language, not excepting even Haig's voluminous work, has this special field been so thoroughly tilled and yielded so rich a crop of physiological and therapeutic facts of importance to the physician. The work is tinctured throughout with new and original ideas concerning the pathogenesis of many disorders, and the subject matter is presented in such form that the veriest tyro may read and understand. Not only is this volume the latest publication on the subject of uric acid; but, for the use of the general practitioner, the best that we have seen. We do not hesitate to recommend it to every physician who is, or who is not, interested in the subject of uric acid.

---

FIVE HUNDRED SURGICAL SUGGESTIONS.—Practical Brevities in Surgical Diagnosis and Treatment. By Walter M. Brickner, B. S., M. D., Chief of Surgical Department, Mount Sinai Hospital Dispensary, New York; Editor-in-Chief *American Journal of Surgery*, and Eli Moschcowitz, A. B., M. D., Assistant Physician Mount Sinai Hospital Dispensary, New York; Associate Editor *American Journal of Surgery*. Second Series. Duodecimo; 125 pages. New York: Surgery Publishing Co., 92 William St., 1907. Price, $1.

It is not surprising that the first edition of "Surgical Suggestions" was quickly exhausted. The attractive little volume was most favorably received by reviewers, and its contents—the snappy, practical "suggestions"—have been reprinted again and again by medical journals all over the country.

In this second series all the surgical suggestions of the first issue have been incorporated and as many more, making a total of five hundred terse, useful "therapeutic hints and diagnostic wrinkles." Several new topics have been thus introduced and the old ones much expanded.

We warmly commend this book. Those wearied by searching for information through ponderous text-books and lengthy articles will find actual refreshment in Surgical Suggestions, everyone of the five hundred paragraphs of which is a separate and useful bit of practical knowledge.

